# Preoperative Suspicion of Internal Hernia and Laparoscopic Repair of a Congenital Transmesocolic Hernia Presenting as Closed-Loop Small-Bowel Obstruction: A Case Report

**DOI:** 10.70352/scrj.cr.26-0516

**Published:** 2026-09-25

**Authors:** Rumi Shimano, Hideaki Murase, Hideaki Iseki

**Affiliations:** Department of Surgery, Tokyo Metropolitan Hiroo Hospital, Tokyo, Japan

**Keywords:** internal hernia, transmesocolic hernia, small-bowel obstruction, laparoscopy, congenital mesocolic defect

## Abstract

**INTRODUCTION:**

Transmesocolic hernia is a rare cause of small-bowel obstruction, and preoperative diagnosis remains difficult because of nonspecific clinical and radiologic findings.

**CASE PRESENTATION:**

A 69-year-old woman presented with abdominal pain and vomiting. Contrast-enhanced CT demonstrated a closed-loop small-bowel obstruction adjacent to the ligament of Treitz, raising suspicion of an internal hernia. Emergency laparoscopic exploration revealed incarceration of a 12-cm segment of jejunum through a transverse mesocolic defect. The bowel was successfully reduced laparoscopically without resection, and the defect was closed using a continuous barbed suture. Direct laparoscopic inspection demonstrated no communication between the defect and the lesser sac, suggesting that the incarcerated bowel remained confined within the transverse mesocolon. The postoperative course was uneventful, and the patient was discharged on POD 8.

**CONCLUSIONS:**

This case highlights the value of early laparoscopic intervention for transmesocolic hernia and provides additional insight into the anatomical variability of transverse transmesocolic hernias.

## INTRODUCTION

Internal hernias are a rare cause of small-bowel obstruction, but they may lead to bowel strangulation and ischemia if diagnosis is delayed.^1)^ Transmesocolic hernia is an uncommon subtype in which the small bowel herniates through a defect in the mesocolon, and transverse transmesocolic hernia is particularly rare in adults.^1,2)^ Preoperative diagnosis remains challenging because clinical manifestations and radiologic findings are often nonspecific.^3,4)^ We report a case of transverse transmesocolic hernia presenting as a closed-loop small-bowel obstruction that was successfully treated laparoscopically without bowel resection. The case was notable for an unusual anatomical configuration without communication with the lesser sac.

## CASE PRESENTATION

A 69-year-old woman with a history of appendectomy, inguinal hernia repair, and hysterectomy with left oophorectomy was transferred to our hospital for worsening small-bowel obstruction. Two days before surgery, she developed abdominal pain and vomiting and was admitted to a referring hospital. Nasogastric decompression was initiated for suspected small-bowel obstruction; however, her abdominal pain worsened the following day. Because emergency surgical treatment was not available at the referring institution, she was transferred to our hospital.

Physical examination revealed abdominal distension and tenderness without muscular guarding or rebound tenderness. Laboratory tests showed no significant inflammatory response.

Abdominal radiography demonstrated dilated small-bowel loops with multiple air-fluid levels, consistent with small-bowel obstruction. Contrast-enhanced CT revealed clustered, dilated small-bowel loops in a closed-loop configuration, with the transition point estimated to be approximately 80 cm distal to the ligament of Treitz, suggesting an internal hernia (**Fig. 1A**, **1B**). Neither the whirl sign nor mesenteric vessel crowding was identified. Although internal herniation was suspected preoperatively, the exact type and location of the hernia could not be determined.

**Fig. 1:**
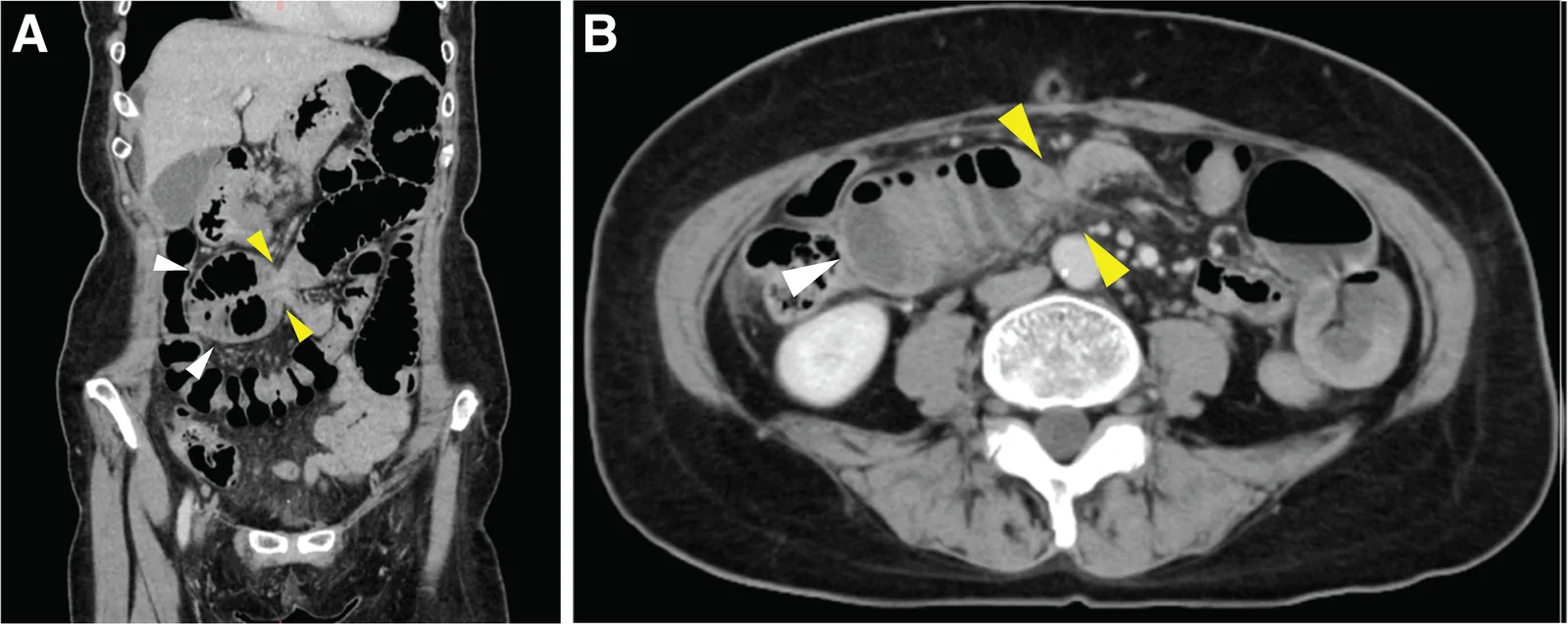
Preoperative contrast-enhanced CT findings. (**A**) Coronal CT image showing clustered, dilated small-bowel loops with a closed-loop configuration. (**B**) Axial CT image demonstrating clustered bowel loops suggestive of an internal hernia. In both panels, yellow arrowheads indicate the mesocolic defect (hernia orifice), and white arrowheads indicate the incarcerated bowel loops.

Emergency laparoscopic exploration was performed because of a suspected strangulating small-bowel obstruction. A 12-mm camera port was inserted through the umbilicus, and three 5-mm working ports were placed in the right mid-abdomen, left mid-abdomen, and left lower abdomen.

Intraoperatively, a 12-cm segment of jejunum was found incarcerated through a defect in the transverse mesocolon located near the ligament of Treitz (**Fig. 2A**). The mesocolic defect measured approximately 2 × 3 cm. After reduction of the incarcerated bowel, direct inspection of the defect revealed no communication with the lesser sac (**Fig. 2B**; **Video S1**). The defect was identified on the posterior aspect of the transverse mesocolon, and the herniated bowel appeared to remain confined within the transverse mesocolon without entering the lesser sac.

**Fig. 2:**
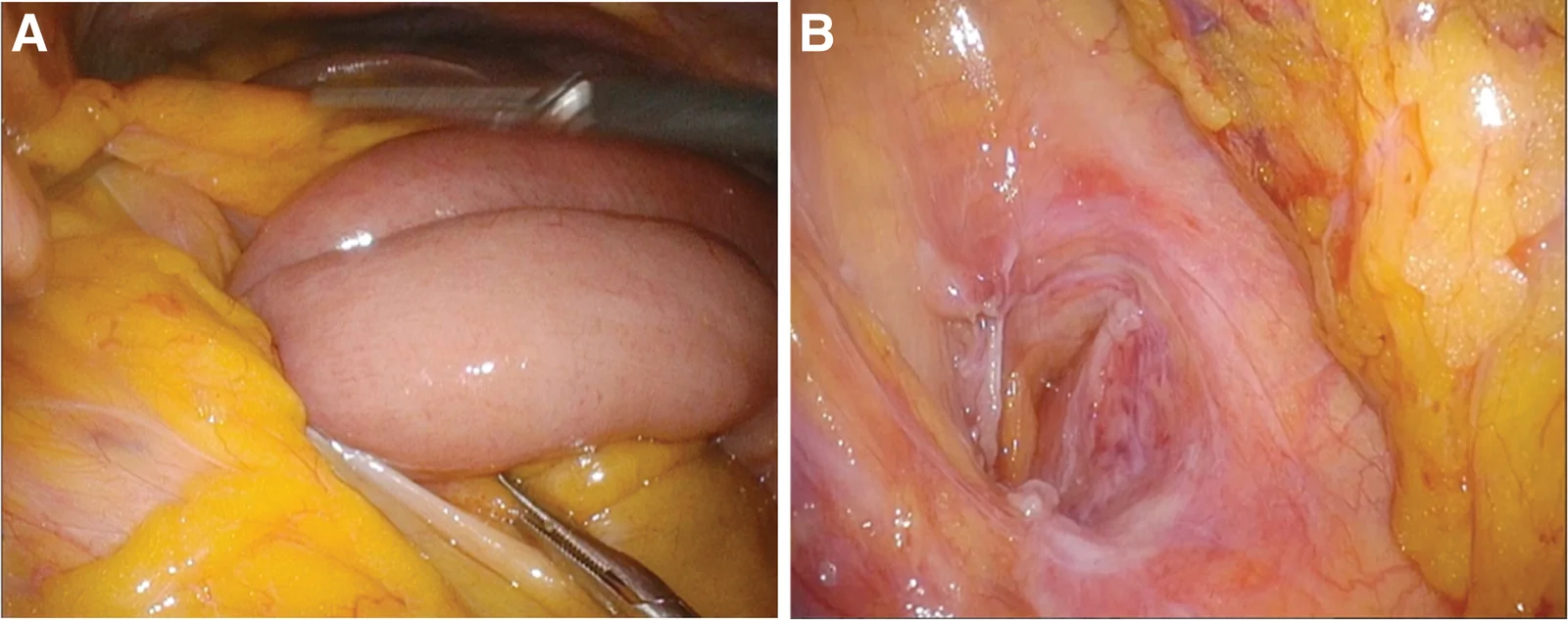
(**A**) A jejunal loop incarcerated through a defect in the transverse mesocolon. (**B**) Direct laparoscopic inspection of the mesocolic defect demonstrating no communication with the lesser sac.

Following reduction, the jejunum showed normal color and preserved peristalsis without evidence of ischemia or necrosis; therefore, bowel resection was not required. A small seromuscular injury of the jejunum occurred during reduction and was repaired through a mini-laparotomy. The mesocolic defect was closed intracorporeally using a continuous 3-0 barbed suture (V-Loc; Medtronic, Minneapolis, MN, USA). The operative time was 116 minutes, and blood loss was negligible.

The patient recovered uneventfully. Oral intake was resumed on POD 3, and she was discharged on POD 8. No complications or recurrence have been observed during 6 months of follow-up.

## DISCUSSION

Internal hernias are an uncommon cause of small-bowel obstruction and remain an important diagnostic challenge because delayed treatment may result in bowel strangulation and ischemia.^1)^ Among them, transmesocolic hernia is a particularly rare subtype, and preoperative identification of the specific anatomical defect remains difficult despite advances in CT.^1,3,4)^ Consequently, many cases are diagnosed only during emergency surgery.^1,3)^

In the present case, CT demonstrated a localized closed-loop obstruction immediately adjacent to the ligament of Treitz. Although classical CT findings suggestive of strangulation, such as the whirl sign or mesenteric vessel crowding, were absent, the unusual location of the closed-loop segment raised suspicion for an internal hernia.^2,3)^ Emergency laparoscopy subsequently confirmed incarceration of a jejunal segment through a transverse mesocolic defect. The absence of bowel ischemia on CT was consistent with the intraoperative findings and likely contributed to the favorable outcome without bowel resection.

Internal hernias occurring near the ligament of Treitz should be differentiated from left paraduodenal and transomental hernias, although preoperative diagnosis is often difficult. Left paraduodenal hernia typically demonstrates clustered small-bowel loops in the left upper abdomen caused by herniation through Landzert’s fossa, whereas transomental hernia results from a defect in the greater or lesser omentum and may show bowel loops within the lesser sac with beak or whirl signs on CT. Recognition of these characteristic imaging features may aid in distinguishing transverse transmesocolic hernia from these entities, although definitive diagnosis often depends on intraoperative findings.^1,5)^

To better characterize this rare entity, we reviewed English-language adult case reports of congenital transverse transmesocolic hernia published since 2000 (**Table 1**).^2,6–24)^ Neonatal cases, clearly iatrogenic defects, non-English publications, reports in which the anatomical location could not be confirmed as the transverse mesocolon, and reports describing congenital paraduodenal (middle mesocolic) hernia based on rotational anomalies rather than a transverse mesocolic defect were excluded. Although the etiology of transverse transmesocolic defects remains incompletely understood, most included cases were considered congenital because no causative abdominal surgery or trauma involving the transverse mesocolon was identified. In our patient, previous appendectomy, inguinal hernia repair, and hysterectomy with left oophorectomy were anatomically unrelated to the transverse mesocolon, and no postoperative adhesions, inflammatory changes, or traumatic findings were identified around the defect. Therefore, a congenital defect was considered the most likely etiology. However, although no direct evidence of postoperative traction was identified, it is conceivable that chronic traction associated with postoperative omental or mesenteric adhesions may have enlarged a pre-existing congenital weakness over time. Therefore, an acquired modifying mechanism cannot be completely excluded.

**Table 1:** Adult cases of congenital transverse transmesocolic hernia reported in the English-language literature since 2000

Author	Year	Age/sex	Previous surgery	Key CT findings	Bowel viability	Approach	Lesser sac
Fujiwara et al.^2)^	2000	65/F	Hysterectomy	Lesser sac herniation	Stenosis (resection)	Open	Yes
Chou et al.^6)^	2005	17/F	None	Combined transmesocolic–transomental hernia	Cyanotic, viable	Open	Yes
Tauro et al. (Case 1)^7)^	2007	19/F	None	Strangulated ileum	Necrosis (resection)	Open	No
Tauro et al. (Case 2)^7)^	2007	25/M	None	Not performed	Viable	Open	No
Liu et al.^8)^	2008	48/F	None	Lesser sac herniation	Ischemia (resection)	Open	Yes
Hashimoto et al. (Case B)^9)^	2012	19/F	None	Closed-loop obstruction	Viable	Open	No
Edwards and Al-Tayar^10)^	2013	32/M	None	Encapsulated small bowel	Viable	Open	No
Alaker and Mathias^11)^	2014	55/F	None	Whirlpool sign	Viable (volvulus)	Open	No
Kundaragi et al.^12)^	2014	55/M	None	Combined transmesocolic–transomental hernia	Viable	Open	Yes
Kassir et al.^13)^	2014	44/F	None	Transmesocolic hernia	Viable	Conservative	No
Subasinghe et al.^14)^	2015	48/F	Cesarean section	Not performed	Viable	Open	Yes
Kishiki et al.^15)^	2015	61/M	None	Clustered small bowel	Viable	Laparoscopic	No
Moudgil et al.^16)^	2015	14/M	None	Not performed	Viable (volvulus)	Open	Yes
Danial et al.^17)^	2019	25/F	None	Not performed	Viable	Laparoscopic converted	No
Aparício et al. (Case 1)^18)^	2019	40/M	None	Sigmoid volvulus	Necrosis (resection)	Open	No
Aparício et al. (Case 2)^18)^	2019	92/F	None	Sigmoid volvulus	Necrosis (resection)	Open	Yes
Kulkarni et al.^19)^	2020	54/F	Hysterectomy	Combined transmesocolic–transomental hernia	Viable	Laparoscopic	Yes
Beh et al.^20)^	2020	93/F	None	Sigmoid volvulus	Necrosis (resection)	Laparoscopic converted	No
Nolasco Vaz et al.^21)^	2021	77/F	Cholecystectomy	Clustered small bowel	Viable	Open	Yes
Kurtkulağı and Çelik^22)^	2024	74/F	None	Mesenteric vascular distortion	Small perforation	Open	No
Shrestha et al.^23)^	2025	71/F	None	Transition point	Viable	Open	Yes
Inoue et al.^24)^	2025	50/M	None	Mesenteric vessel convergence	Congested, viable	Laparoscopic	No
Present case	2025	69/F	No colonic surgery	Closed-loop obstruction	Viable	Laparoscopic	No

The table summarizes patient characteristics, preoperative imaging findings, operative approach, bowel viability, presumed etiology, and the anatomical relationship between the mesocolic defect and the lesser sac. Cases were included only when the defect was confirmed to originate from the transverse mesocolon. Communication with the lesser sac was determined from operative descriptions or intraoperative images whenever possible.

F, female; M, male

Laparoscopic management of congenital transverse transmesocolic hernia remains uncommon but is increasingly reported.^15,21,24)^ In our review, laparoscopic surgery was attempted in 5 patients, including 3 completed laparoscopically and 2 requiring conversion to laparotomy because of limited working space or inadequate visualization. These findings suggest that laparoscopy is a feasible diagnostic and therapeutic approach in selected patients when bowel viability is preserved, while prompt conversion should not be hesitated when adequate visualization or safe bowel reduction cannot be achieved. Recently, Inoue et al. successfully treated an idiopathic transverse mesocolic hernia laparoscopically without bowel resection, further supporting the feasibility of a minimally invasive approach in appropriately selected patients.^24)^ When reducing incarcerated bowel through a narrow mesocolic defect, excessive traction on the edematous bowel wall should be avoided whenever possible. Gentle traction on the adjacent mesentery is preferable, and when direct bowel manipulation is unavoidable, grasping the bowel through a gauze cushion may help distribute traction pressure and minimize serosal injury. In addition, surgeons should not hesitate to enlarge the hernia orifice by careful incision when safe reduction cannot be achieved by gentle traction alone. In the present case, a minor seromuscular injury occurred during reduction, underscoring the importance of these technical considerations, particularly when the incarcerated bowel is edematous and the hernia orifice is tight.

The most distinctive finding in the present case was the absence of communication between the mesocolic defect and the lesser sac. Direct laparoscopic inspection confirmed that the defect did not communicate with the lesser sac, suggesting that the incarcerated jejunum remained confined within the transverse mesocolon rather than entering the lesser sac. A schematic illustration of the proposed anatomical configuration is shown in **Fig. 3**. As illustrated in **Fig. 3A**, the hernia orifice was located in the transverse mesocolon near the ligament of Treitz. **Figure 3B** illustrates the proposed anatomical relationship. Intraoperatively, the hernia orifice was identified on the posterior aspect of the transverse mesocolon and did not communicate with the lesser sac. These findings suggest that the jejunum entered through a defect in the posterior leaf and remained between the mesocolic layers. However, the precise relationship between the incarcerated bowel and the anterior leaf of the transverse mesocolon could not be confirmed intraoperatively. Therefore, definitive classification as a true intramesocolic hernia was not possible. In contrast, several previously reported cases described herniation into the lesser sac through the mesocolic defect. As summarized in **Table 1**, among 22 reviewed cases and the present case in which the relationship between the hernia and the lesser sac could be determined, 10 (43.5%) communicated with the lesser sac, whereas 13 (56.5%) did not. These findings indicate that communication with the lesser sac is not a consistent anatomical feature of congenital transverse transmesocolic hernia and support the concept that this entity comprises heterogeneous anatomical patterns. Recognition of this variability may facilitate a more accurate anatomical understanding of this rare condition and may help explain differences in operative findings among reported cases. In the present case, the precise relationship between the mesocolic defect and the middle colic artery could not be determined with certainty because the preoperative CT was not obtained during the arterial phase, the markedly dilated bowel obscured the mesenteric vascular anatomy, and this anatomical relationship was not specifically documented intraoperatively.

**Fig. 3:**
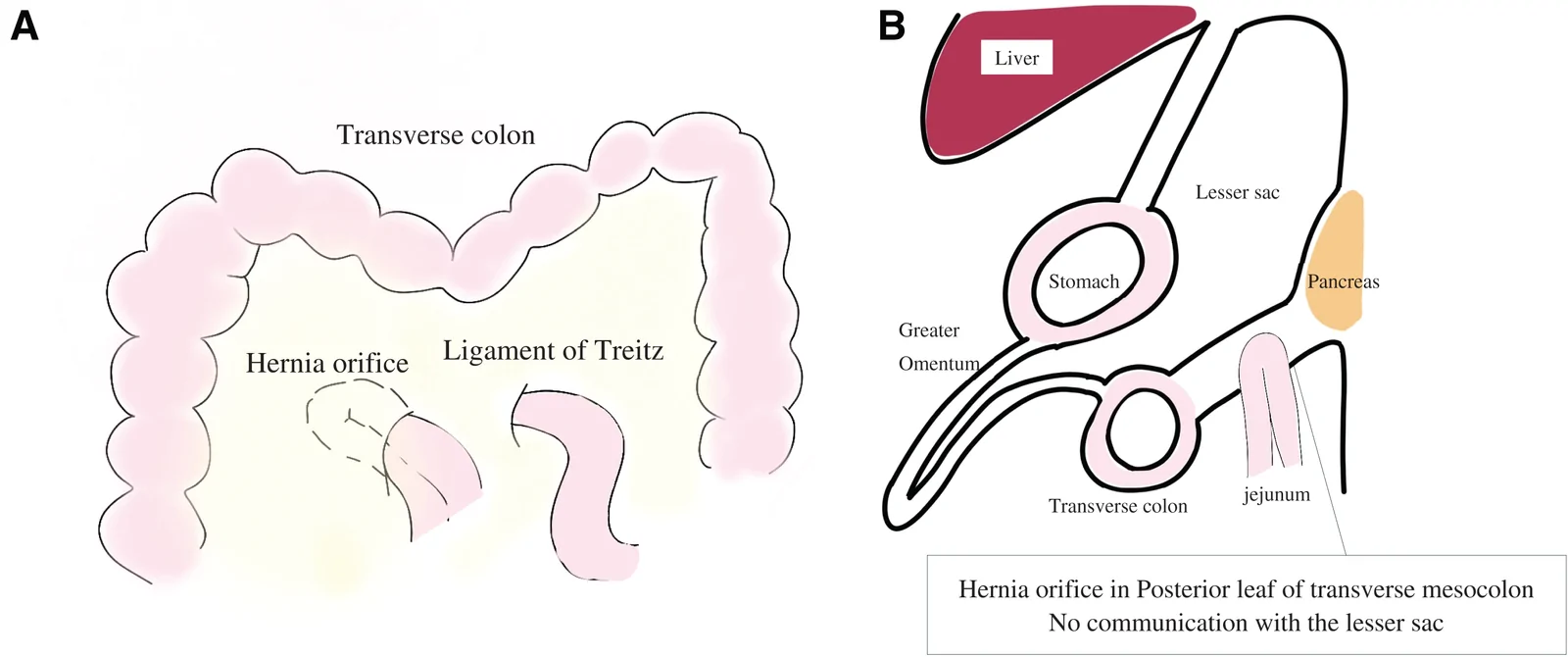
Schematic illustration of the proposed anatomical configuration in the present case. (**A**) Coronal schematic showing the approximate location of the hernia orifice in the transverse mesocolon near the ligament of Treitz. (**B**) Sagittal schematic illustrating herniation of the jejunum through a defect in the posterior leaf of the transverse mesocolon. The incarcerated bowel remained confined within the transverse mesocolon without communication with the lesser sac.

This case has several important clinical implications. First, transverse transmesocolic hernia should be considered in patients presenting with a closed-loop obstruction near the ligament of Treitz, even in the absence of classical CT signs of strangulation. Second, early surgical exploration before the development of bowel ischemia increases the likelihood of bowel preservation and successful minimally invasive management. Finally, careful intraoperative assessment of the relationship between the mesocolic defect and the lesser sac may provide important anatomical information that is not apparent on preoperative imaging and contributes to a better understanding of the anatomical spectrum of transverse transmesocolic hernias.

## CONCLUSIONS

Congenital transmesocolic hernia should be considered in patients presenting with closed-loop small-bowel obstruction near the ligament of Treitz. Early laparoscopic exploration may facilitate bowel preservation, and careful intraoperative assessment can improve understanding of the anatomical spectrum of this rare condition.

## SUPPLEMENTARY MATERIALS

Video S1 Laparoscopic management of a transverse transmesocolic hernia. The video demonstrates incarceration of the jejunum through a transverse mesocolic defect, reduction of the herniated bowel, direct inspection of the defect, which shows no communication with the lesser sac, and intracorporeal closure of the defect using a 3-0 barbed suture (V-Loc; Medtronic, Minneapolis, MN, USA).

## DECLARATIONS

### Availability of data and materials

Data sharing is not applicable to this article because no datasets were generated or analyzed during the current study.

## References

[1] Martin LC, Merkle EM, Thompson WM. Review of internal hernias: radiographic and clinical findings. AJR Am J Roentgenol 2006; 186: 703–17. 10.2214/AJR.05.0644 16498098

[2] Fujiwara T, Ohno Y, Sasaki A, et al. Internal hernia with triple hiatus of congenital origin: report of a case. Surg Today 2000; 30: 954–8. 10.1007/s005950070054 11059742

[3] Lanzetta MM, Masserelli A, Addeo G, et al. Internal hernias: a difficult diagnostic challenge. Review of CT signs and clinical findings. Acta Biomed 2019; 90: 20–37. 10.23750/abm.v90i5-S.8344 PMC6625567 31085971

[4] Blachar A, Federle MP, Dodson SF. Internal hernia: clinical and imaging findings in 17 patients with emphasis on CT criteria1. Radiology 2001; 218: 68–74. 10.1148/radiology.218.1.r01ja5368 11152781

[5] Nguyen BH, McGuirt AS. Radiographic features of a strangulated transomental hernia. Cureus 2024; 16: e62484. 10.7759/cureus.62484 PMC11251898 39022475

[6] Chou CK, Mak CW, Wu RH, et al. Combined transmesocolic-transomental internal hernia. AJR Am J Roentgenol 2005; 184: 1532–4. 10.2214/ajr.184.5.01841532 15855111

[7] Tauro LF, Vijaya G, D’Souza CR, et al. Mesocolic hernia: an unusual internal hernia. Saudi J Gastroenterol 2007; 13: 141–3. 10.4103/1319-3767.33468 19858633

[8] Liu ZY, Wang Y, Liang CH. Lesser sac herniation through a defect in the transverse mesocolon: CT findings. Br J Radiol 2008; 81: e50–2. 10.1259/bjr/20661338 18238915

[9] Hashimoto D, Hirota M, Sakata K, et al. Adult transmesenteric hernia: report of two cases. Surg Today 2012; 42: 489–92. 10.1007/s00595-011-0035-2 22075661

[10] Edwards HM, Al-Tayar H. A transmesenteric congenital internal hernia presenting in an adult. J Surg Case Rep 2013; 2013: rjt099. 10.1093/jscr/rjt099 PMC3887995 24968431

[11] Alaker M, Mathias J. Internal herniation through a defect in the transverse mesocolon. BMJ Case Rep 2014; 2014: bcr2013202753. 10.1136/bcr-2013-202753 PMC3939399 24567183

[12] Kundaragi NG, Vinayagam S, Mudali S. Stretched bowel sign in combined transmesocolic and transomental internal hernia: a case report and review of literature. Indian J Radiol Imaging 2014; 24: 171–4. 10.4103/0971-3026.134407 PMC4094971 25024528

[13] Kassir R, Boutet C, Blanc P, et al. Transmesocolic hernia without a history of digestive surgery: unknown disease. J Curr Surg 2014; 4: 55–7.

[14] Subasinghe D, Keppetiyagama CT, Samarasekera DN. Jejunal obstruction due to a variant of transmesocolic hernia: a rare presentation of an acute abdomen. BMC Surg 2015; 15: 57. 10.1186/s12893-015-0051-z PMC4431459 25953110

[15] Kishiki T, Mori T, Hashimoto Y, et al. Laparoscopic repair of internal transmesocolic hernia of transverse colon. Case Rep Surg 2015; 2015: 853297. 10.1155/2015/853297 PMC4515260 26246930

[16] Moudgil A, Pandove PK, Singh A, et al. An unusual case of transverse mesocolic internal hernia with abnormality of both hands and high arched feet. Int J Surg Case Rep 2015; 6: 226–9. 10.1016/j.ijscr.2014.10.040 PMC4334881 25553528

[17] Danial AK, Alsaman MZB, Zazo R, et al. A unique case of congenital transmesocolic hernia with malrotation in a 25-year-old female: a case report. J Surg Case Rep 2019; 2019: rjz280. 10.1093/jscr/rjz280 PMC6796187 31636890

[18] Aparício DJ, Leichsenring C, Pignatelli N, et al. Transmesocolic hernia with sigmoid colon strangulation without surgical history: a series of two case reports. J Surg Case Rep 2019; 2019: rjz073. 10.1093/jscr/rjz073 PMC6425156 30906521

[19] Kulkarni GV, Premchandani D, Chitnis A, et al. Laparoscopic repair of a combined transmesocolic, transomental hernia. J Minim Access Surg 2020; 16: 83–6. 10.4103/jmas.JMAS_244_18 PMC6945348 30777991

[20] Beh HN, Ongso YF, Koong DB. Transmesocolon internal hernia masking as simple sigmoid volvulus. J Surg Case Rep 2020; 2020: rjaa017. 10.1093/jscr/rjaa017 PMC7061819 32175071

[21] Nolasco Vaz J, Dias R, Faria A, et al. Transverse congenital transmesocolic hernia: a case report and review of the literature. Clin Surg 2021; 6: 3082.

[22] Kurtkulağı Ö, Çelik AO. Transverse mesocolic internal hernia: a case of acute small bowel obstruction. Int Surg J 2024; 11: 1858–60.

[23] Shrestha S, Maharjan S, Bhatta BR, et al. Intestinal obstruction in a patient with congenital transverse mesocolic defect with internal hernia: a case report. Int J Surg Case Rep 2025; 129: 111121. 10.1016/j.ijscr.2025.111121 PMC11930737 40058219

[24] Inoue D, Tokuhara K, Matsuki H, et al. Internal hernia through an idiopathic transverse mesocolon defect treated by laparoscopic surgery: a case report. Int J Surg Case Rep 2025; 134: 111701. 10.1016/j.ijscr.2025.111701 PMC12328845 40729804

